# Effect of Castration Type and Diet on Growth Performance, Serum Sex Hormones and Metabolites, and Carcass Quality of Heavy Male Pigs

**DOI:** 10.3390/ani12081004

**Published:** 2022-04-13

**Authors:** Leticia Pérez-Ciria, Francisco Javier Miana-Mena, Javier Álvarez-Rodríguez, Maria Angeles Latorre

**Affiliations:** 1 Departamento de Producción Animal y Ciencia de los Alimentos, Instituto Agroalimentario de Aragón-IA2, Universidad de Zaragoza-CITA, C/Miguel Servet 177, 50013 Zaragoza, Spain; leticiapcgm@gmail.com; 2 Departamento de Farmacología y Fisiología, Instituto Agroalimentario de Aragón-IA2, Universidad de Zaragoza-CITA, C/Miguel Servet 177, 50013 Zaragoza, Spain; jmiana@unizar.es; 3 Departamento de Ciencia Animal, Universidad de Lleida, Av. Alcalde Rovira Roure 191, 25198 Lleida, Spain; javier.alvarez@udl.cat

**Keywords:** male pigs, immunocastration, dietary energy, dietary protein, growth performance, sex hormones, serum metabolites, carcass quality

## Abstract

**Simple Summary:**

Castration is indispensable in male pigs intended for the production of high-quality dry-cured ham; however, for welfare reasons, alternatives are being sought and, among them, immunocastration stands out. The literature shows that immunocastration might generate lower fat deposition than surgical castration, which is undesirable for the dry-curing process. Therefore, it would be interesting to confirm the impact of immunocastration in male pigs and to test feeding plans that benefit fatness. The aim of this trial was to assess the effect of type of castration (surgical vs. immunological) and diet (control vs. high energy vs. low crude protein and amino acids) on growth performance, serum sex hormones and metabolites, and carcass quality of male pigs destined for the Protected Designation of Origin Teruel ham. Although immunocastration improved productive performance, it reduced carcass fatness. Also, alternative diets to control improved the feed conversion ratio but did not influence carcass quality traits. In conclusion, under a quality point of view, surgical castration would be preferable over immunocastration for dry-cured ham production, and the use of moderately high-energy or low-crude-protein and -amino-acids diets from 80 to 137 kg of body weight would not provide improvements.

**Abstract:**

A trial was carried out to study the effect of type of castration and diet on pigs destined for Teruel ham production, which is a Spanish protected designation of origin for dry-cured ham. A total of 144 Duroc × (Landrace × Large White) male pigs were used. Half of them were surgically castrated and the other half were immunocastrated with three doses at approximately 25, 58 and 79 kg of body weight. Furthermore, three diets (control vs. high energy vs. low crude protein-CP- and amino acids-AA) were tested from 80 to 137 kg of body weight. Growth performance, serum sex hormones and metabolites, and carcass quality were evaluated. Immunocastrated males grew faster and had better feed conversion ratio than surgically castrated males, but presented lower carcass fatness. Pigs fed the high-energy diet and the low-CP and -AA diet were more efficient at transforming feed into gain than those fed the control diet, but no effect was detected on carcass quality. In conclusion, surgically castrated males are preferable than immunocastrated males for Teruel dry-cured ham elaboration. Besides, a high-energy diet or a low-CP and -AA diet might improve productive performances, but does not provide any benefit in terms of carcass quality.

## 1. Introduction

The protected designation of origin (PDO) “Teruel ham” is a Spanish PDO for the production of high quality dry-cured hams from crossbred pigs (not autochthonous). To guarantee their quality, the Consortium requires, among other aspects [1], pigs slaughtered with heavy body weight (BW) (at least 86 kg of carcass weight) and fatty (fat depth measured over the gluteus medius muscle (GM) greater than 16 mm). Then, animals reach sexual maturity and, in the case of males, the castration becomes essential to avoid boar taint in the pork and, in fact, male castration is other requirement of this PDO. Traditionally, it is performed by surgical methods but, for welfare reasons, alternatives are sought, among which is immunocastration. This practice usually consists in the application of two doses of a gonadotrophin releasing factor (GnRF) analogue protein conjugate. The first dose only primes the pig’s immune system, and the second one stimulates the production of a large quantity of antibodies against GnRF, neutralizing the pig’s endogenous GnRF and reducing testicular function [2]. The consequences of immunocastration in males on body fat retention are not clear, but some studies [3,4,5] show that it might generate leaner carcasses than surgical castration. It would be a handicap for the elaboration of Teruel ham, taking into account that a considerable fat thickness is required to avoid an excessive curing of the pieces [6]. However, no sufficiently robust experiment to assess immunocastration in males destined to that type of dry-cured ham has been developed. For this reason, it would be interesting to study the impact of immunocastration of these males and the feeding plans for them that could benefit body fat retention. In this regard, the increase of the energy content [7,8] and the decrease of the crude protein (CP) and amino acids (AA) levels [9,10] in the diet have been related to increases of body fat accretion. Therefore, the aim of the current trial was to evaluate the effect of the type of castration (surgical vs. immunological) and the feeding provided (control diet vs. high-energy diet vs. low-CP and -AA diet) on growth performance, serum sex hormones and metabolites, and carcass quality of male pigs destined for Teruel dry-cured ham elaboration.

## 2. Materials and Methods

### 2.1. Animals Husbandry and Experimental Design

A total of 144 Duroc × (Landrace × Large White) male pigs were used. Half of them were surgically castrated during the first week of life. The other half received the first dose for immunocastration (Improvac^®^, Zoetis Belgium SA, Louvain-la-Neuve, Belgium) at the end of the post-weaning period, with 56 ± 3 days of age (around 25 kg BW), upon request of the PDO Teruel ham Consortium. At the age of 78 ± 3 days, pigs were moved to the fattening farm (Teruel, Spain), individually identified with ear tags, weighed (35.3 ± 4.10 kg BW) and group-penned (eight pigs/pen; space allowance 1.1 m^2^/animal). Each pen had a 50% slatted floor, a grow feeder, and a drinking cup. Animals were allotted to blocks of increasing BW and each block contained all treatments. A second dose of Improvac^®^ at 57.7 ± 5.60 kg BW (101 ± 3 days of age) and a third dose at 79.2 ± 7.20 kg BW (122 ± 3 days of age) to ensure the effect of this product were injected to the pigs previously vaccinated.

Three experimental diets were provided to both surgically castrated males (SCM) and immunocastrated males (IM) during the grower and finisher periods. The grower phase lasted from 122 to 149 ± 3 days of age (around 80–110 kg BW) and the finisher phase lasted from 150 ± 3 days of age to the day before slaughter (around 110–137 kg BW). The tested diets were the same as those used in a previous study about immunocastration in gilts [11]; (i) a control diet with a nutritional profile similar to the recommendations of FEDNA [12]; (ii) a diet with a higher energy content than the control diet, with similar CP and AA percentages; and (iii) a diet with lower CP and AA contents than the control diet, with the same energy level. In all experimental feeds, the ideal protein concept was maintained [12] and the change between the grower and the finisher feeds was carried out on a fixed day. Table 1 shows the analyzed nutrient composition of the diets. More information about them (ingredients, estimated nutritional value and a more complete description of the analyzed nutrients) as well as the methods used to analyze the chemical composition are reported in Pérez-Ciria et al. [11]. Feeding in pellet form and water were provided ad libitum throughout the trial. Therefore, there were six experimental treatments: two types of castration (surgical vs. immunological) × three diets (control vs. high energy vs. low CP and AA).

### 2.2. Performance Measurements

Individual BW was measured at several times: on the arrival at the fattening farm (22 days after the first dose of Improvac^®^), when the second dose was administered, when the third dose was applied (coinciding with the day in which pigs received the experimental grower diets), the first supply day of the experimental finisher diets, and the day before slaughter. Data of BW were used to calculate average daily gain (ADG) per pen. During the grower and finisher periods, feed consumption per pen was recorded in order to calculate average daily feed intake (ADFI). Average daily gain and ADFI were used to estimate the feed conversion ratio (FCR).

### 2.3. Blood Sampling and Analyses

Ten milliliters of blood from one representative pig per pen, always the same and close to the average BW, were taken around 10–12 a.m. at several times: when the second and third immunocastration doses were injected, at the end of the grower period and at the end of the finisher period (coinciding with the previous day of slaughter). More information about blood sampling and processing until its analysis is described in Pérez-Ciria et al. [11].

In the case of sex hormones, the concentrations of testosterone and estradiol were determined in the samples taken in the days of application of the second and third immunization doses and also the day before slaughter using competitive immunoassays with enzyme-labeled chemiluminescent technology (IMMULITE, Siemens Healthineers, Madrid, Spain).

The concentrations of albumin and urea, as protein biomarkers, and triglycerides and cholesterol, as lipid biomarkers, were determined in the samples taken at the end of both the grower and finisher periods. For it, the same methods reported in Pérez-Ciria et al. [11] were followed using the same equipment (GernonStar equipment, RAL S.A., Barcelona, Spain).

### 2.4. Slaughtering and Carcass Measures

The slaughter BW target was 135–140 kg, and thus loads were programmed in three times; with 178, 185, and 199 ± 3 days of age, achieving 137 kg BW on average. Pigs were slaughtered after a fasting period of 13 h in a commercial abattoir (Teruel, Spain) located 130 km from the farm. In the current trial, a total of 102 animals were chosen at random for the study of carcass quality, 17 from each experimental treatment (type of castration × diet). The following measures were recorded of each pig: hot carcass weight, fat depth over the GM, ham length, ham perimeter and ham and shoulder weights. Later, carcass yield, total (ham + shoulder) weight and ham, shoulder and total yields were calculated. Details about slaughter and measurements taken on the carcass are described in Pérez-Ciria et al. [11].

### 2.5. Statistical Analyses

Data were analyzed with the Statistical Analysis System (SAS Institute Inc., Cary, NC, USA). Growth performance parameters were assessed as a randomized complete block design with treatments arranged factorially (2 × 3) using the GLM procedure. Serum sex hormones and metabolites were analyzed by repeated-measures analysis using the MIXED procedure. The covariance structures chosen were: variance components for testosterone, albumin, urea and cholesterol; heterogeneous autoregressive for estradiol; and compound symmetry for triglycerides. Carcass quality was analyzed as a factorial design (2 × 3) using the GLM procedure. The pig was the experimental unit, except in the case of growth performance parameters, which was the pen. Tukey test was utilized for pairwise comparison of the least square means. More complete information about statistical analyses is described in Pérez-Ciria et al. [11], where the same procedures were used. A *p*-value < 0.05 was considered as significant and a *p*-value between 0.05 and 0.10 as a tendency.

## 3. Results and Discussion

Except for serum testosterone and cholesterol concentrations, the remaining results are presented as main effects, as no significant interactions were found.

### 3.1. Growth Performance

From the beginning of the trial (22 days post-1st dose) to the second dose of Improvac^®^ (a total of 23 days), the IM tended (*p* = 0.056) to grow slower than SCM (Table 2). Batorek et al. [13] already reported a similar response in a meta-analysis. In this period, IM are physiologically similar to entire males, since the first dose of immunocastration only primes the pig’s immune system [2]. The lower ADG in IM than in SCM might be due to a lower feed intake; it was not recorded in the present trial but it has been observed by other authors [14,15]. This effect has also been found when entire males and SCM were compared [16,17,18], which could be related to higher levels of testicular hormones in entire males than in SCM [19]. Cronin et al. [20] have reported more aggressive and sexual behaviors in entire males, which would reduce their eating times. Between the second and the third dose of immunization against GnRF (a total of 21 days), the differences in ADG between IM and SCM disappeared (*p* = 0.828). According to the manufacturer of Improvac^®^, within one week post-2nd dose, an induction of anti-GnRF antibodies can be expected, and that leads to a reduction of aggressive and sexual behavior around one to two weeks post-2nd vaccination. However, from the third dose to the day before slaughter (a total of 56, 63 or 77 days, depending on the day of slaughter), IM grew faster (*p* = 0.0004), tended (*p* = 0.063) to eat more feed, and had lower (*p* < 0.0001) FCR than SCM. This improvement in growth rate observed in IM in comparison to SCM has been supported by a great deal of reports [13,21,22]. Batorek et al. [23] and Brunius et al. [19] observed that IM exhibited greater serum insulin-like growth factor-1 concentration than SCM at three weeks post-2nd dose and one day prior to slaughter, respectively. Therefore, IM would have a higher anabolic potential than SCM [19], contributing to improved feed efficiency and causing faster growth [23]. In addition, Morales et al. [15] suggested that the extra growth detected in IM after the second vaccination might result from a compensatory response to the reduced growth after the first injection. Regarding the increase of feed intake in IM, it should be noted that the levels of serum testosterone begin to drop after the second dose, which might increase the appetite in these animals. Also, Batorek et al. [23] found that IM presented relatively low amounts of serum leptin compared with SCM at 24 days post-2nd vaccination, and it is known that leptin reduces appetite [24]. For the overall trial (from 22 days post-1st dose to the day before slaughter), IM grew faster (*p* = 0.007) than SCM, although it did not have any influence (*p* = 0.337) on the time the pigs stayed on the farm.

For the overall period in which the diets were tested (from around 80 to 137 kg BW), ADG was similar for all groups (*p* = 0.439). There is some unanimity in the literature [8,25,26] about the lack of effect of increasing dietary energy concentration on ADG in heavy pigs. This finding could be explained because the growth rate of pigs fed the high-energy level could be limited by the less lysine intake [27]. However, it is worth noting that, although just numerically, pigs given the high energy diet ate less feed (*p* = 0.10) than the other groups, this being especially observed during the grower period (from around 80 to 110 kg BW; *p* = 0.04). The negative relationship between dietary energy level and feed consumption in pigs has also been found by other authors [8,28], and it is justified because pigs adjust their voluntary feed intake to maintain a constant daily energy intake [29]. Regardless the lack of effect of low-CP and -AA diet on ADG and ADFI in comparison to the control diet, it corroborates the results of Knowles et al. [25], although Suárez-Belloch et al. [30] reported a lower ADG and ADFI when a greater reduction of dietary CP and AA was applied (from 17.2 to 10.6% CP and from 0.77 to 0.42% lysine). Furthermore, the high-energy diet and also the low-CP and -AA diet generated lower (*p* = 0.007) FCR than the control diet, which was more pronounced in the finisher period (from around 110 to 137 kg BW; *p* = 0.022). It confirms the results of a previous trial performed by our team using the same diets in gilts [11]. The better results in feed efficiency obtained with a high energy diet in comparison to a standard (control) diet agree with Suarez-Belloch et al. [8], but disagree with Knowles et al. [25], and it could be due to the different nutrient levels tested, feed ingredients, genetic types, target slaughter BW and/or experimental conditions. The better FCR in animals fed the low-CP and -AA diet vs. the control diet was not expected. Applying a greater CP and AA restriction, Suárez-Belloch et al. [30] obtained the opposite effect. This could indicate that, in the present study, the low-CP and -AA diet did not limit growth, and therefore, the CP and AA contents of the control diet could be overestimated. The results obtained with the experimental diets would imply that the increase in 0.15 Mcal of net energy/kg in the diet, from 80 to 137 kg BW, in barrows intended for Teruel dry-cured ham elaboration, could be beneficial for pig farmers, depending on the price of fat sources. In addition, in this period and in this type of animals, a reduction in 2% of CP and 0.10% of standardized ileal digestible lysine would decrease feeding costs, increase the profits of pig farmers and reduce nitrogen losses to the environment [31].

### 3.2. Serum Sex Hormones

**Figure 1:**
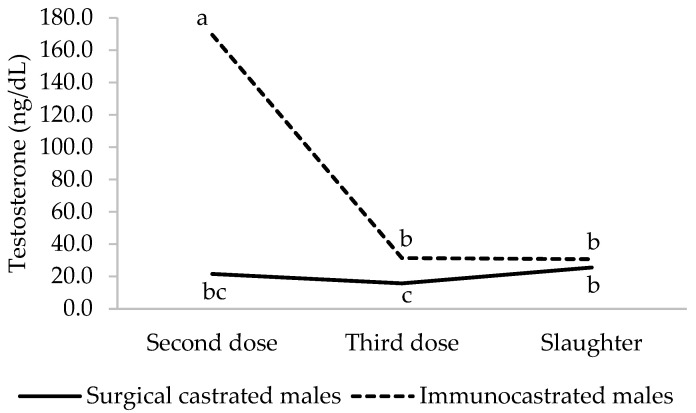
Interaction type of castration × sampling time (*p* < 0.0001) on serum testosterone concentration (*n* = 9 per type of castration at each sampling time). After the data had been transformed, statistical analysis was performed on it. Data are presented as back-transformed least square means. Different letters (a, b or c) are used to indicate values that differ significantly (*p* < 0.05).

Testosterone concentration in serum was higher in IM than in SCM when the second dose of Improvac^®^ was administered (*p* < 0.001) (Figure 1). These differences were also detected, but numerically much lower, when the third dose was applied (*p* = 0.0006). It was expected because only 21 days had passed. The day before slaughter, no impact of the type of castration was detected on testosterone levels (*p* = 0.903). Han et al. [32] and Yamsakul et al. [33] also observed that IM showed higher serum testosterone concentration than SCM when the second dose was injected, and Zamaratskaia et al. [34] found it in plasma just before the second dose injection. Testosterone concentration began to drop abruptly, in the present study, after the second dose, resulting in no significant difference between male groups the day before slaughter, which is in line with a great deal of reports [19,32,34]. The reason is that luteinizing hormone levels decrease quickly after the second dose [35], and this hormone stimulates testosterone production in Leidig cells [36]. According to Claus et al. [35], the subsequent gradual decrease of testosterone levels in IM seems to be due to the clearance of testosterone stored in adipose tissues, becoming measurable in the blood.

Regarding estradiol levels in serum (Table 3), it was greater (*p* = 0.034) in IM than in SCM (31.3 vs. 24.3 pg/mL, respectively), irrespective of the sampling time (*p* = 0.801 for the interaction type of castration × sampling time). Despite this, immunocastration seemed to reduce estradiol secretion, taking into account that Han et al. [37] observed that entire male pigs presented around 70 pg of estradiol/mL of serum between the second dose and the slaughter. Also, in the current study, the estradiol concentration was maintained from the second to the third application and increased from that moment to the slaughter (*p* = 0.0002). The results found about this parameter in the literature are quite variable. Han et al. [37] detected higher estradiol concentrations in IM than in SCM when the second dose was injected but no difference at slaughter, and Van den Broeke et al. [38] did not find differences between IM and SCM at any time point.

### 3.3. Serum Metabolites

The effect of pig male immunocastration on serum metabolites has been scarcely investigated. In the current trial, no impact of it was detected (*p* > 0.10) on albumin, urea or triglycerides levels (Table 4). The present results would suggest that gonadal steroids suppression did not have carryover effects on nutritional status, as the main blood protein (albumin), dietary protein breakdown marker (urea), and lipid anabolism marker (tryglicerides), were kept similar.

The type of diet did not affect either albumin concentration, which is in agreement with Pérez-Ciria et al. [11] who evaluated similar feeds in heavy gilts. Kim et al. [39] also obtained the same results when comparing diets with different energy level. Nevertheless, Mule et al. [40] and Suárez-Belloch et al. [41] found that, applying a higher and earlier CP and AA restriction, albumin levels decreased. It could be explained because, with low-CP and -AA diets, the limitations in the availability of AA would firstly appear in the synthesis of exported proteins, such as albumin [42].

However, diet did influence urea concentration; pigs fed the low-CP and -AA diet presented lower (*p* = 0.008) urea levels than those fed the control diet, with pigs fed the high-energy diet being in an intermediate position. The similar urea content from control and high-energy diets is consistent with the findings of other authors [11,39,43]. Also, the lower urea content from low-CP and -AA diet agrees with several reports [30,40,44] who compared diets with different CP and AA contents. It would indicate that the utilization of low-CP and -AA diets reduces the degradation of protein and AA [45], improving the AA utilization for growth [46], which would reduce the urinary nitrogen excretion, having important implications to reduce manure load.

Furthermore, animals eating the high-energy diet presented greater (*p* = 0.039) triglyceride levels than those eating the low-CP and -AA diet, with the animals eating the control diet in an intermediate place. These results confirm those of Kim et al. [39] and Suárez-Belloch et al. [30], although in another study, Suárez-Belloch et al. [41] observed that an earlier (at 26 kg BW) and greater dietary CP and AA restriction (from 24 to 14.8% CP and from 1.1 to 0.52% lysine) promoted a linear increase of serum triglycerides. It is worth noting that the higher triglyceride concentration obtained with the high-energy diet suggests that the administration of a high-energy diet could be a good strategy to increase lipid biosynthesis in pigs.

**Figure 2:**
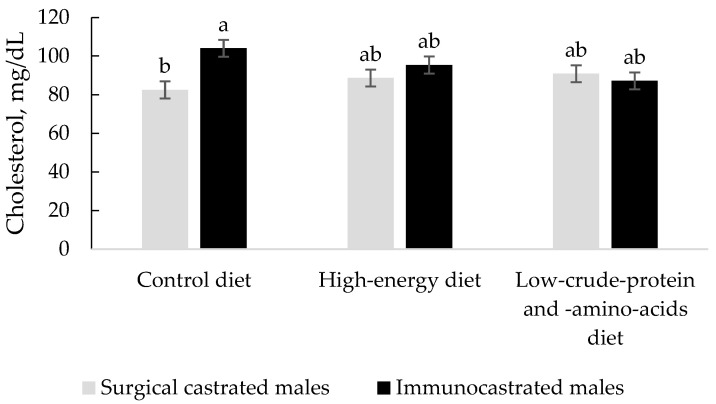
Interaction type of castration × diet (*p* = 0.032) on serum cholesterol concentration (*n* = 6). Least square means that differ significantly (*p* < 0.05) are noted with different letters (a, b).

In the present trial, a significant interaction between type of castration and diet was detected for serum cholesterol concentration (*p* = 0.032, Figure 2); whereas the cholesterol content was similar for IM and SCM providing the high-energy diet or the low-CP and -AA diet, IM showed higher cholesterol levels than SCM when the control diet was given. Therefore, when providing the high-energy diet or the low-CP and -AA diet, the differences between IM and SCM would be minimized regarding cholesterol levels. The higher serum cholesterol concentration observed in IM fed the control diet could be a negative aspect if the same results were obtained in their tissues, but Harris et al. [47] found that the amount of cholesterol accretion in tissues was not generally influenced by the serum cholesterol concentration of the animal. Cholesterol can be obtained from the diet if it contains animal fat sources. However, in this study, the dietary fat was palm oil, which implies that the outcomes reflect only endogenous synthesis. The lower serum cholesterol in SCM compared to IM fed the control diet would suggest that its hepatic synthesis was downregulated.

With regard to the sampling time, albumin, urea and cholesterol levels were higher (*p* < 0.0001, *p* = 0.014 and *p* < 0.0001, respectively) in the finisher period than in the grower period, which corroborates the results reported previously by Pérez-Ciria et al. [11] with the same diets in gilts. It means that these metabolites increase as pigs are older or heavier irrespective of feeding and sex.

### 3.4. Carcass Quality

The influence of the type of castration and diet on carcass quality can be found in Table 5. The slaughter weight had been pre-fixed and then the BW at slaughter was similar (*p* = 0.158) in animals from both types of castration. However, IM presented lower carcass weight (*p* = 0.003) and carcass yield (*p* = 0.006) than SCM. The lower carcass yield observed in IM agrees with other studies [15,48,49], and it could be mainly due to the weight of the testicles and to the higher weight of the full intestinal tract, liver, kidneys and additional male reproductive tract reported in IM in comparison to SCM [50]. Also, fat thickness over the GM was thinner (*p* = 0.0004) in IM than in SCM, which is consistent with the results of Gispert et al. [51], Morales et al. [14] and Škrlep et al. [3] and not positive for dry-cured ham elaboration. Other authors [48,49] did not find it significant, but numerically observed the same effect. The lower carcass fatness detected in IM, in the current study, was also observed in the intramuscular fat content of the pork of these animals [52]. This is because IM are similar to entire males until immunocastration is effective, shortly after the second dose, and later they behave as castrated males [53], while SCM are barrows from the first week of life. Therefore, the shorter the time interval between the second dose of immunocastration and slaughter, the less fatness could the IM be expected to present [54]. No impact (*p* > 0.10) of type of castration was detected on ham size, in agreement with Pinna et al. [55] and Daza et al. [56]. However, hams from IM were lighter (*p* = 0.0009) than those from SCM, which would be related to the lighter weight of IM carcasses. On the other side, no influence of type of castration was observed on ham yield in carcass (*p* = 0.222), corroborating the findings of Morales et al. [14,15], although other authors [48,49] observed higher ham yield in IM. No differences between IM and SCM were found in shoulder weight (*p* = 0.313), but shoulder yield was greater (*p* = 0.0007) in IM than in SCM, which agrees with the reports of Lowe et al. [57] and Pauly et al. [58]. The discrepancies between studies in the yield of main lean cuts may be due to the different immunocastration protocols (period between the last dose and slaughter), breed or practices of trimming (more or less amount of fat removed in the pieces).

Despite having pre-fixed the target slaughter weight, there was a slight difference of around 5 kg between the BW mean of pigs fed the high-energy diet and of pigs fed the control diet, resulting in a significant difference between them (*p* = 0.009). However, carcass weight and also carcass yield were similar for all dietary treatments (*p* > 0.10). Several authors reported similar carcass weights by increasing dietary energy [7,8,27] or decreasing CP and lysine of the diet [59,60,61], although Wood et al. [62] did observe that pigs fed low-CP and -AA levels had lighter carcasses than those fed high levels. Regarding carcass yield, some reports [26,27] show a linear increase as dietary energy content increased, which could be explained because low-energy diets have more fiber content, and an increase in fiber intake increases gastrointestinal tract weight, reducing carcass yield [63]. In the present study, the difference in fiber content between the control and the high-energy diet was not enough to carry out a significant increase of the gastrointestinal tract weight. Also, no influence of diet was found on fat depth over the GM (*p* = 0.191). Suarez-Belloch et al. [8] did observe that fat depth measured at that point was greater with the increase of the dietary energy (in base on fat from animal origin), and Sirtori et al. [64] detected this effect with the lowest CP and AA diet, which could be attributed to the fact that excess of energy intake in relation to lysine intake is transformed into body fat [65,66]. Weight and yield of ham and shoulder were not affected by the feedstuffs (*p* > 0.10). In the current work, the lack of effect on carcass fatness and also perhaps to proportions of the main lean cuts could be due in part to the high variability of data, but probably more to the mild differences in nutrient levels of the tested diets.

## 4. Conclusions

It can be concluded that SCM would be preferable to IM for Teruel dry-cured ham production, because surgical castration increases carcass fatness, although the slower growth and worse feed conversion ratio in these animals should be considered. On the other hand, increasing dietary energy by 0.15 Mcal of net energy/kg or decreasing dietary CP by 2% and AA, against a standard diet, in male pigs from 80 to 137 kg BW and regardless of the type of castration, seems to be interesting, since they improve feed efficiency but, from a carcass quality perspective, no benefit is noted.

## Figures and Tables

**Table 1:** Analyzed nutrient composition of the experimental diets (%, as-fed basis).

Item	Grower Diet (80 to 110 kg BW)			Finisher Diet (110 to 137 kg BW)		
	Control	High Energy	Low CP-AA ^1^	Control	High Energy	Low CP-AA ^1^
Gross energy, Mcal/kg	3.99	4.12	3.92	3.91	4.12	3.95
Crude protein	16.2	15.9	14.4	14.5	15.1	12.7
Ether extract	3.55	5.88	3.44	3.00	5.65	3.73
Neutral detergent fiber	10.9	10.2	10.5	10.5	8.96	10.2
Total lysine	0.98	0.98	0.79	0.76	0.77	0.71
Total methionine	0.28	0.27	0.25	0.24	0.25	0.23
Total threonine	0.62	0.60	0.59	0.56	0.58	0.51

BW: body weight. ^1^ low in crude protein and amino acids.

**Table 2:** Impact of type of castration and diet on growth performance (least square means) of heavy male pigs.

Item ^1^	Type of Castration		SEM ^2^ (*n* = 9)	Diet			SEM ^2^ (*n* = 6)	*p*-Value ^4^	
	Surgical	Immunological		Control	High Energy	Low CP-AA ^3^		Type of Castration	Diet
Body weight, kg									
Initial ^5^	36.0	34.7	0.29	35.1	35.3	35.7	0.35	0.011	0.448
Second dose	59.7	57.6	0.37	58.2	58.9	58.9	0.46	0.002	0.528
Start grower period (third dose)	81.5	79.2	0.83	79.7	80.9	80.5	1.01	0.074	0.697
Start finisher period	109.4	109.6	0.86	110.0	108.7	109.8	1.05	0.886	0.662
Day before slaughter ^6^	136.1 (133.1–139.1)	138.4 (135.4–141.5)	-	135.7 (132.1–139.5)	138.1 (134.4–141.9)	137.9 (134.2–141.6)	-	0.262	0.565
ADG Initial-2nd dose, kg/day	1.032	0.994	0.0130	1.007	1.025	1.008	0.0159	0.056	0.679
ADG 2nd-3rd dose, kg/day	1.038	1.030	0.0279	1.023	1.050	1.029	0.0342	0.828	0.838
Grower period ^7^									
ADG, kg/day	0.995	1.084	0.0282	1.081	0.991	1.047	0.0346	0.046	0.224
ADFI, kg/day	3.30	3.35	0.061	3.47 ^a^	3.17 ^b^	3.33 ^ab^	0.074	0.578	0.040
FCR	3.32	3.10	0.059	3.22	3.20	3.21	0.072	0.020	0.984
Finisher period ^8^									
ADG, kg/day	0.860	1.001	0.0220	0.888	0.950	0.954	0.0269	0.0007	0.194
ADFI, kg/day	3.24	3.53	0.077	3.43	3.27	3.47	0.094	0.020	0.319
FCR	3.78	3.53	0.071	3.87 ^a^	3.47 ^b^	3.64 ^ab^	0.087	0.030	0.022
Overall diets period ^9^									
ADG, kg/day	0.927	1.040	0.0164	0.981	0.966	1.003	0.0200	0.0004	0.439
ADFI, kg/day	3.27	3.44	0.058	3.44	3.22	3.40	0.072	0.063	0.100
FCR	3.53	3.31	0.027	3.52 ^a^	3.34 ^b^	3.39 ^b^	0.033	<0.0001	0.007
Overall trial period ^10^									
ADG, kg/day	0.974	1.026	0.0115	0.996	0.997	1.008	0.0141	0.007	0.795
Length, day	184.2	182.7	1.10	182.7	185.0	182.7	1.35	0.337	0.397

^1^ ADG: average daily gain; ADFI: average daily feed intake; FCR: feed conversion ratio. ^2^ SEM: standard error of the mean. ^3^ low in crude protein and amino acids. ^4^ No significant interactions (type of castration × diet) were found (*p* > 0.05). ^5^ Twenty two days post-first dose. ^6^ After the data had been transformed, statistical analysis was performed on it. Data are presented as back-transformed least square means and 95% confidence intervals within parentheses. ^7^ From the third dose to around 110 kg BW. ^8^ From around 110 kg BW to the day before slaughter. ^9^ From the third dose to the slaughter or when the experimental diets were tested. ^10^ From the entrance to the fattening farm (22 days post-1st dose) to the day before slaughter. Least square means within a row with different superscript (^a,b^) differ (*p* < 0.05).

**Table 3:** Impact of type of castration and sampling time on the serum estradiol concentration (least square means) of heavy male pigs.

Item	Estradiol, pg/mL
Type of castration	
Surgical	24.3
Immunological	31.3
SEM ^1^ (*n* = 27)	2.22
Sampling time	
At second dose of immunocastration	24.6 ^b^
At third dose of immunocastration	24.2 ^b^
Day before slaughter	34.6 ^a^
SEM ^1^ (*n* = 18)	1.93
*p*-value ^2^	
Type of castration	0.034
Sampling time	0.0002

^1^ SEM: standard error of the mean. ^2^ No significant interaction (type of castration × sampling time) was found (*p* = 0.801). Least square means within a column with different superscript (^a,b^) differ (*p* < 0.05).

**Table 4:** Impact of type of castration, diet and sampling time on serum metabolites (least square means) of heavy male pigs.

Item	Albumin ^1^, g/dL	Urea, mg/dL	Triglycerides, mg/dL
Type of castration			
Surgical	4.06 (3.89–4.24)	33.9	48.1
Immunological	4.00 (3.81–4.18)	31.9	46.4
SEM ^2^ (*n* = 18)	-	1.59	2.91
Diet			
Control	4.05 (3.82–4.29)	37.7 ^a^	45.1 ^ab^
High energy	4.01 (3.80–4.23)	32.7 ^ab^	55.6 ^a^
Low crude protein and amino acids	4.02 (3.81–4.24)	28.4 ^b^	41.2 ^b^
SEM ^2^ (*n* = 12)	-	1.95	3.55
Sampling time			
At the end of the grower period	3.35 (3.18–3.51)	30.0	52.1
At the end of the finisher period	4.77 (4.58–4.97)	35.8	42.5
SEM ^2^ (*n* = 18)	-	1.59	4.27
*p*-value ^3^			
Type of castration	0.611	0.378	0.686
Diet	0.964	0.008	0.039
Sampling time	<0.0001	0.014	0.217

^1^ After the data had been transformed, statistical analysis was performed on it. Data are presented as back-transformed least square means and 95% confidence intervals within parentheses. ^2^ SEM: standard error of the mean. ^3^ No significant interactions (type of castration × diet, type of castration × sampling time, diet × sampling time, type of castration × diet × sampling time) were found (*p* > 0.05). Least square means within a column with different superscript (^a,b^) differ (*p* < 0.05).

**Table 5:** Impact of type of castration and diet on carcass quality (least square means) of heavy male pigs.

Trait	Type of Castration (C)		SEM ^1^ (*n* = 51)	Diet (D)			SEM ^1^ (*n* = 34)	*p*-Value ^3^	
	Surgical	Immunological		Control	High Energy	Low CP-AA ^2^		C	D
Slaughter weight, kg	136.1	138.6	1.26	134.1 ^b^	140.8 ^a^	137.2 ^ab^	1.53	0.158	0.009
Carcass									
Weight, kg	106.1	103.6	0.56	104.7	105.5	104.3	0.69	0.003	0.491
Yield, %	77.1	75.6	0.38	76.1	76.8	76.2	0.47	0.006	0.559
Fatness over the GM ^4,5^, mm	23.9	20.6	0.64	21.4	23.4	21.9	0.79	0.0004	0.191
Ham ^5^									
Length, cm	39.8	39.7	0.16	39.7	39.8	39.8	0.20	0.515	0.882
Perimeter, cm	79.0	78.4	0.26	78.9	78.9	78.4	0.32	0.109	0.400
Main cut weight ^5^, kg									
Ham ^6^	13.7 (13.5–13.9)	13.3 (13.1–13.4)	-	13.5 (13.3–13.7)	13.5 (13.3–13.8)	13.3 (13.1–13.6)	-	0.0009	0.380
Shoulder	9.04	9.12	0.060	9.19	9.04	9.01	0.074	0.313	0.195
Total ^7^	22.7	22.3	0.12	22.6	22.5	22.3	0.15	0.066	0.319
Main cut yield ^5^, % carcass									
Ham ^6^	12.9 (12.7–13.1)	12.7 (12.5–12.9)	-	12.8 (12.6–13.0)	12.8 (12.5–13.0)	12.8 (12.5–13.0)	-	0.222	0.892
Shoulder	8.52	8.82	0.061	8.78	8.59	8.63	0.074	0.0007	0.186
Total ^6,7^	21.4 (21.1–21.6)	21.5 (21.3–21.8)	-	21.6 (21.2–21.9)	21.4 (21.1–21.7)	21.4 (21.1–2.7)	-	0.352	0.671

^1^ SEM: standard error of the mean. ^2^ low in crude protein and amino acids. ^3^ No significant interactions (type of castration × diet) were found (*p* > 0.05). ^4^ GM: gluteus medius muscle. ^5^ From the left side of the carcass. ^6^ After the data had been transformed, a statistical analysis was performed on it. Data are presented as back-transformed least square means and 95% confidence intervals within parentheses. ^7^ Ham and shoulder. Least square means within a row with different superscript (^a,b^) differ (*p* < 0.05).

## Data Availability

Data can be available on request.
